# Impact of Responsible AI on the Occurrence and Resolution of Ethical Issues: Protocol for a Scoping Review

**DOI:** 10.2196/52349

**Published:** 2024-06-05

**Authors:** Selina Boege, Madison Milne-Ives, Edward Meinert

**Affiliations:** 1 Centre for Health Technology School of Nursing and Midwifery University of Plymouth Plymouth United Kingdom; 2 Translational and Clinical Research Institute Newcastle University Newcastle-upon-Tyne United Kingdom; 3 Department of Primary Care and Public Health School of Public Health Imperial College London London United Kingdom; 4 Faculty of Life Sciences and Medicine King's College London London United Kingdom

**Keywords:** artificial intelligence, AI, responsible artificial intelligence, RAI, ethical artificial intelligence, trustworthy artificial intelligence, explainable artificial intelligence, XAI, digital ethics

## Abstract

**Background:**

Responsible artificial intelligence (RAI) emphasizes the use of ethical frameworks implementing accountability, responsibility, and transparency to address concerns in the deployment and use of artificial intelligence (AI) technologies, including privacy, autonomy, self-determination, bias, and transparency. Standards are under development to guide the support and implementation of AI given these considerations.

**Objective:**

The purpose of this review is to provide an overview of current research evidence and knowledge gaps regarding the implementation of RAI principles and the occurrence and resolution of ethical issues within AI systems.

**Methods:**

A scoping review following Preferred Reporting Items for Systematic Reviews and Meta-Analyses Extension for Scoping Reviews (PRISMA-ScR) guidelines was proposed. PubMed, ERIC, Scopus, IEEE Xplore, EBSCO, Web of Science, ACM Digital Library, and ProQuest (Arts and Humanities) will be systematically searched for articles published since 2013 that examine RAI principles and ethical concerns within AI. Eligibility assessment will be conducted independently and coded data will be analyzed along themes and stratified across discipline-specific literature.

**Results:**

The results will be included in the full scoping review, which is expected to start in June 2024 and completed for the submission of publication by the end of 2024.

**Conclusions:**

This scoping review will summarize the state of evidence and provide an overview of its impact, as well as strengths, weaknesses, and gaps in research implementing RAI principles. The review may also reveal discipline-specific concerns, priorities, and proposed solutions to the concerns. It will thereby identify priority areas that should be the focus of future regulatory options available, connecting theoretical aspects of ethical requirements for principles with practical solutions.

**International Registered Report Identifier (IRRID):**

PRR1-10.2196/52349

## Introduction

### Background

The development of artificial intelligence (AI) is expanding across industries and its implementation has created ethical apprehensions, centered on the responsible creation and use of AI systems. Various guidelines, regulations, and frameworks have emerged with the goal of delivering responsible application and progression of AI [1]. The notable increase in instances of ethical harm has been caused by the misuse of technology (eg, voter manipulation, facial recognition surveillance, and unauthorized mass data collection) or by design flaws in the technology itself (eg, bias in recidivism, loan rejection, and medical misdiagnosis) [2]. Other ethical concerns include the transparent and equitable usage of data, safeguarding data privacy across platforms and systems, ensuring data availability and quality, endorsing data integration for interoperability, and addressing intellectual property concerns [1]. Responsible AI (RAI) promotes the adoption of ethical frameworks and the incorporation of RAI’s 3 main pillars of accountability (ie, the need to explain and validate decisions), responsibility (ie, users interpreting results and identifying errors), and transparency (ie, the need to describe, examine, and duplicate mechanisms) to foster moral responsibility in emerging technology [3].

### Rationale

AI ethics is an emerging field that falls within the broader domain of digital ethics and addresses concerns arising from the development and implementation of new digital technologies [4]. Due to AI’s transformative potential, the discourse of guiding values and principles for its development and implementation highlights the need for resolving concerns for AI for data privacy, accountability, and inadvertently fostering bias. Governance against unregulated uses, privacy violations, and algorithm biases requires resilience for security and the preparation for potential attacks. By delivering and maintaining accessibility, safety, accuracy, and fairness, these challenges can be addressed through standardized RAI principles that involve ethical consideration and stakeholder participation [4-8].

The collaboration among diverse stakeholders in developing RAI principles and policies demonstrates the potential for developing robust ethical guidance. RAI principles could have a tangible impact on reducing both the frequency and intensity of ethical issues that arise in various contexts. The implementation of RAI principles exerts a positive influence on the overall ethical landscape, leading to a discernible decrease in the occurrence of ethical dilemmas and mitigating the severity of those that do emerge. The adoption of RAI principles could foster a more accountable AI ecosystem with heightened attention to conducting responsible research and innovation [8].

Recent literature advocates for continued efforts to adhere to RAI principles as a means of fostering a more ethical and trustworthy AI landscape and suggests the incorporation of RAI principles as a vital safeguard, not only in curbing the frequency of ethical issues but also in minimizing their impact. Academia, government, and corporate institutions have published guidelines aimed at fostering the responsible development and deployment of AI technology, encompassing beneficence, nonmaleficence, autonomy, justice or fairness, and accountability, as crucial considerations in ensuring RAI practices [2,9,10]. Various entities, both public and private, offer diverse RAI frameworks, contributing to democratization but lacking consensus and standardization in ethical values. Practical support for AI practitioners is limited, with few frameworks covering all phases of the software development life cycle (SDLC), which ensures the completion of all functionalities, user requirements, objectives, and end goals when creating, planning, and maintaining the development of a software project. The SDLC enhances the overall quality of software programs and the software development process and establishes a structured approach that comprises 7 distinct stages of planning, requirement gathering and analysis, design, coding, testing, deployment, and maintenance [8]. Theoretical frameworks are currently deficient in practical assistance encompassing all SDLC phases in developing, testing, and deploying RAI applications, including practical validation techniques for theoretical principles, as well as supporting stakeholders during the implementation and auditing phases. The synthesis of positive effects of RAI mitigation strategies through the application of RAI principles for minimizing ethical harm and the lack of standardization reveal the shortcomings of complete, uniform, and user-friendly RAI frameworks in current literature. Research value can be added that supports stakeholders across the entire SDLC and is accessible to different stakeholders (both technical and nontechnical), is streamlined, and expedites the adoption of RAI practices [8]. There is a need for both an update and an overview of effective and standardized RAI frameworks to reveal the current state of RAI principles driving positive change and mitigating ethical risks [2,9-14].

Given the evidence of the feasible effectiveness of RAI mitigation strategies for ethical issues in a broader sense, it is necessary to develop a comprehensive and multidisciplinary approach throughout the AI pipeline by embracing a practical and cohesive approach to RAI implementation [14-21]. Current literature on RAI primarily focuses on ethical principles like transparency and fairness but lacks detailed guidance on their practical implementation across the AI development lifecycle [19]. The main gap in the literature lies in transitioning from dynamic and changing theoretical frameworks to actionable strategies for embedding ethical principles in real-world AI applications [19].

### Aim and Research Questions

This scoping review will offer an overview and summarize the state of the field of ethical issues inherent to AI systems, linked RAI practices, their strengths and weaknesses, the studies evaluating them, and gaps in the literature. This will help to inform directions of the present ethical issues and current practices to reduce the occurrence and resolve them through RAI principles (Table 1). This study aims to assess the impact of RAI principles on resolving ethical challenges inherent to AI systems. This review will be based on our research question: How does the use of RAI practices and ethical frameworks impact the occurrence and resolution of ethical issues within AI systems?

**Table 1:** Types of ethical issues and responding RAI^a^ principles [2].

Ethical issue (categorized)	Description	Linked RAI code (categorized)
Bias or discrimination	AI^b^ systems can exhibit bias and discriminate against individuals or groups based on factors such as race, gender, or socioeconomic status. This can lead to unfair treatment and perpetuate existing inequalities	Principle of fairness and nondiscrimination: ensuring that AI systems do not exhibit bias or discriminate against individuals or groups based on protected characteristics
Lack of transparency and explainability	AI systems often operate as black boxes, hindering understanding of their decision-making process and raising concerns about biases and errors	Principle of XAI^c^: techniques such as interpretable machine learning, model explanations, and rule-based systems are used to provide insights into how AI systems arrive at their decisions, increasing transparency
Privacy and data protection	AI systems rely on personal data, necessitating safeguards and measures to address privacy breaches and unauthorized use	Principle of privacy: using privacy-enhancing technologies, anonymization techniques, data minimization practices, and secure data handling protocols to protect individuals’ privacy rights and prevent unauthorized access or misuse of personal data
Accountability and responsibility	AI systems can have unintended consequences and significant socioeconomic impacts, requiring comprehensive assessments and proactive measures to mitigate risks and address socioeconomic effects	Principle of auditing: conducting rigorous risk assessments, including ethical impact assessments, can help identify and mitigate potential risks and unintended consequences of AI systems. This may involve using frameworks such as the AI Ethics Impact Assessment Tool kit and involving multidisciplinary teams to evaluate the societal, environmental, and economic implications of AI applications

^a^RAI: responsible artificial intelligence.

^b^AI: artificial intelligence.

^c^XAI: explainable artificial intelligence.

## Methods

### Overview of the Study Design

Scoping reviews represent a specialized form of literature review that focuses on charting available literature across a wide-ranging subject area. Scoping reviews are well-suited for outlining present research pinpointing essential insights and areas of understanding that warrant deeper investigation [22]. The Preferred Reporting Items for Systematic Reviews and Meta-Analyses Extension for Scoping Reviews (PRISMA-ScR) and Population, Intervention, Comparator, Outcome, and Studies (PICOS) framework were used to build the search strategy (Textbox 1) and provide a methodological framework for the scoping review.

**Population, intervention, comparator, outcome, and studies (PICOS) framework.**

**Population**

Organizations developing artificial intelligence (AI) systems.

**Intervention**

Implementation of ethical frameworks and responsible artificial intelligence (RAI) principles in practice.

**Comparator**

Not applicable.

**Outcomes**

The focus is to examine the impact of RAI principles on ethical issues in AI systems. Secondary goals involve assessing the strengths, weaknesses, benefits, and limitations of current RAI principles in resolving ethical concerns.

**Study types**

Qualitative, quantitative, and mixed methods studies and literature that present any form of RAI principles for the implementation and resolution of ethical concerns in AI systems are eligible for inclusion. Protocols, reviews, abstracts, and meta-analyses older than 10 years were excluded from this review.

### Search Strategy

The review will search 8 databases, including, PubMed, CORE, Scopus, IEEE Xplore, EBSCO, Web of Science, the ACM Digital Library, and ProQuest (Arts and Humanities). Based on a preliminary review of the literature, Medical Subject Headings (MeSH) terms and keywords were identified and grouped into 4 categories. They will be put together in the following way when searching the databases: RAI (MeSH OR Keywords) AND Ethical Issues (MeSH OR Keywords; Table 2).

**Table 2:** Search terms.

Category	MeSH^a^	Keywords (in title or abstract)
Ethical issues within AI^b^	Ethical challenges in artificial intelligence	“Artificial Intelligence” OR “ethical challenges in AI” OR “ethical issues in AI” OR “Machine Learning” OR “AI governance”
RAI^c^	Responsible AI OR machine learning OR deep learning	“Responsible AI” OR “RAI principles” OR “supervised deep learning” OR “RAI” OR “supervised machine learning” OR “trustworthy AI” OR “Explainable AI” OR “XAI”
Ethical frameworks	AI ethics OR AI governance	“AI ethics” OR “ethical AI principles” OR “moral frameworks in AI” OR “ethical frameworks in AI”
RAI management	Responsible AI management	“Ethical intervention” OR “Responsible AI Management” OR “RAI management”

^a^MeSH: Medical Subject Headings.

^b^AI: artificial intelligence.

^c^RAI: responsible AI.

### Inclusion Criteria

All literature and research proposing RAI principles will be included and studies presenting theoretical impact and suggestions of ethical frameworks will be identified and analyzed independently as well. Interventions aimed at mitigating ethical issues will be included given that they support all main pillars of RAI principles; no comparator is required, and all study types will be eligible for inclusion.

### Exclusion Criteria

Studies that do not evaluate RAI principles or responses to ethical issues within the development and use of AI systems such as protocols, reviews, and abstracts will be excluded. Studies that are not published in the English language and studies published before 2013 will not be eligible for inclusion. Considering the rapid technological advancements and the evolving consideration of AI ethics and RAI, expectations in literature older than 10 years may not provide the current insights and information required for a relevant analysis in this field [9].

### Screening and Article Selection

Article references will be stored, and duplicates will be removed using the citation management software EndNote X9 (Clarivate). The EndNote X9 search function will also be used to conduct an initial screening of the references based on keywords from the search strategy. The remaining titles and abstracts will be screened, and a full-text review will be conducted by the author (SB) to determine final eligibility.

### Data Extraction

Data will be extracted from included studies into a predeveloped charting list, highlighting the data that will be in focus for this scoping review (see Textbox 2).

**Full data charting list.**

**Title**

Title of publication

**Author**

Full name of first author

**Year**

Year of publication

**Country**

The country in which the study is conducted

**Quality or quantity**

Whether the study entails qualitative, quantitative, or mixed methods research. Added “other” category that may fall outside of categories

**Aims or purpose**

Stated aims and objectives the research seeks to meet

**Source of evidence**

Whether the study is primary or secondary research, evidence synthesis, conference abstract, discussion article, etc

**Study design**

Description of methods and activities

**Type of artificial intelligence (AI)–based technology**

Notes on specific AI systems, that is, machine learning (ML), deep neural network (DNN), clinical decision support system (CDSS), and AI augmentation

**Ethical issues**

Inconclusive evidence, inscrutable evidence, misguided evidence, unfair outcomes, transformative effects, traceability, unjustified actions, and opacity

**Responsible artificial intelligence (RAI) principles**

Respect for human autonomy, prevention of harm, fairness, explicability, and patient privacy

**Level of impact (high, medium, and low)**

Data on evidence of the impact of RAI principles on the resolution of ethical issues

**Probability (high, medium, and low)**

Data on the expected likelihood of successful mitigation

**Benefits of RAI principles**

Notes on the benefits of RAI principles

**Limitations of RAI principles and their impact**

Notes on the theoretical and practical restrictions of RAI guidelines including affected impact

**Strengths and limitations of RAI principles**

Notes on the strengths and weaknesses of the use of RAI principles

### Data Analysis and Synthesis

A descriptive analysis will be used given the expected large variety of study types. A meta-analysis will be attempted based on the outcome measures of the impact and influence of RAI principles on the occurrence and resolution of ethical issues, which may also indicate the primary outcome of the meta-analysis. The meta-analysis will aggregate findings on how RAI principles impact ethical issue management in AI systems, focusing on effectiveness metrics and stakeholder perceptions. Once all data were extracted, we would assess the feasibility of a meta-analysis. Given the expected variety of outcomes, a quantitative meta-analysis is unlikely to be possible, but we will synthesize findings of the differential impacts of various RAI principles on diverse ethical concerns and applications. There is a possibility that this may not be feasible due to the expected heterogeneity of studies. The extracted data will be summarized in a narrative synthesis to bring together findings relating to implementation challenges and successes of RAI principles, their influence on policy making, and trends in ethical AI research. This approach aims to provide insights into the operationalization of RAI principles and highlight areas for future research.

## Results

The results will be included in the full systematic review, which is expected to start in June 2024 and be completed for the submission of publication by the end of 2024.

## Discussion

### Expected Findings

This scoping review will provide an overview of the state of the literature regarding the influence of RAI principles on the resolution of ethical issues within AI systems. This section will use the data extracted from studies to explore the conclusions that can be drawn, the limitations of the scoping review, and key areas for future research. Special focus will be placed on relevant stakeholders involved in the ethical AI ecosystem, and studies investigating interventions for RAI development will be summarized and discussed in a subsection. A summary of the current principles, their strengths and weaknesses, and the studies evaluating them will help to inform the development of functional RAI frameworks for mitigating ethical issues for the progress of AI development and use.

### Strengths and Limitations

A key strengths of the proposed study is its use of the PRISMA-ScR guidelines to ensure transparent and replicable reporting of the review and its interdisciplinary approach. By including data across different disciplines, the review will be able to develop a nuanced understanding of RAI principles and their implementation in various contexts. We expect this to lead to a comprehensive, cross-disciplinary dialog that enriches the AI community’s perspective on ethical considerations.

The limitations of the scoping review will be discussed in detail in the full review; key limitations are expected to include the scope and the use of only 1 main reviewer. Although the broad scope is a strength in that the findings will not be limited to a specific discipline, including a broad scope will increase the difficulty of synthesizing various findings and determining lessons that are generalizable. It also means that, although we have selected a variety of databases to obtain good coverage of the literature for the search, there is a possibility of missing relevant studies meeting our inclusion criteria. Another limitation is that the literature search, screening, and data analysis will be executed by only 1 reviewer, which has an increased risk of overlooking relevant research and potential biases due to the lack of diverse perspectives and independent verification mechanisms. It is not expected to be possible for a second reviewer to conduct an independent screening due to time and resource constraints, although this will be reassessed when conducting the review. Additionally, the research team does not have proficiency in other languages, which will hinder our capability to review any literature beyond data sets available in English.

### Future Directions and Dissemination

Future research could build upon findings from this review by conducting in-depth analyses to identify the gaps in ethical AI frameworks, particularly focusing on stakeholder engagement and interventions for RAI development. Additionally, there is a need for longitudinal studies to assess the effectiveness of emerging principles and interventions in mitigating ethical issues throughout the lifecycle of AI systems. The dissemination plan for this paper will involve publishing the findings in a peer-reviewed journal dedicated to computer sciences, social sciences, philosophy, and ethics. A concise policy brief will be drafted for review to inform and guide regulatory frameworks, ensuring accessibility to legislative audiences. The dissemination will leverage digital platforms, including academic social networks and research repositories, to maximize reach and impact across various sectors.

### Conclusions

This scoping review is expected to provide a vital synthesis of research on RAI principles and guidance for efforts to chart a course for the balanced development of AI systems, where innovation is matched with ethical integrity. The scoping review’s findings will likely prompt the development of more nuanced ethical frameworks for AI, influencing both emerging technologies and policy making by highlighting the importance of aligning AI innovation with ethical responsibility. Future research may focus on creating adaptive, real-time monitoring tools to continuously evaluate and guide the ethical implementation of AI as technologies and societal norms evolve.

## Appendix

Multimedia Appendix 1 Search strings.

Multimedia Appendix 2 Preferred Reporting Items for Systematic Reviews and Meta-Analyses extension for Scoping Reviews (PRISMA-ScR) checklist.

## Abbreviations

AI: artificial intelligence
MeSH: Medical Subject Headings
PICOS: population, intervention, comparator, outcome, and studies
PRISMA-ScR: Preferred Reporting Items for Systematic Reviews and Meta-Analyses Extension for Scoping Reviews
RAI: responsible artificial intelligence
SDLC: software development life cycle

## Data Availability

The authors confirm that the data supporting the findings of this study are available within the paper and appendices (Multimedia Appendices 1 and 2).
