# Supplementary material for: Impact of Responsible AI on the Occurrence and Resolution of Ethical Issues: Protocol for a Scoping Review
Source: JMIR Res Protoc. 2024 Jun 5;13:e52349. doi: 10.2196/52349 (PMC11187507; doi:10.2196/52349)
Supplement: Multimedia Appendix 1 [file resprot_v13i1e52349_app1.docx]

| Database | Search String | Retrieved |
| --- | --- | --- |
| PubMed | ("Artificial intelligence"[MeSH Terms] OR "machine learning"[MeSH Terms] OR " ai " [Title/Abstract] OR "machine learning"[All Fields] OR "deep learning"[Title/Abstract] OR "algorithms"[Title/Abstract] OR "neural network"[Title/Abstract] OR "neural networks"[Title/Abstract] OR "supervised machine learning"[Title/Abstract] OR "unsupervised machine learning"[Title/Abstract] OR "reinforcement learning"[Title/Abstract]) AND "Responsible Artificial Intelligence"[All Fields]) OR "Artificial Intelligence ethics"[Title/Abstract] OR "ethical frameworks"[Title/Abstract] OR "AI ethics"[Title/Abstract] OR "supervised machine learning"[Title/Abstract] OR "trustworthy AI"[Title/Abstract] OR "Explainable AI"[Title/Abstract] OR "Business Ethics"[Title/Abstract] | 1,938 Results |
| ERIC (ProQuest) | “Ethical Issues” OR “Ethical challenges” OR “AI ethics” AND “Responsible Artificial Intelligence” OR “RAI” OR “Artificial Intelligence ethics” OR “ethical frameworks” OR “AI ethics” OR “supervised machine learning” OR “trustworthy AI” OR “Explainable AI” | 198 |
| Scopus | "Ethical issues" AND "ethical challenges" AND "responsible artificial intelligence" OR "trustworthy ai" OR "explainable artificial intelligence" | 100 |
| IEEE Xplore | ("Ethical Issues" OR "Ethical challenges" AND "Responsible Artificial Intelligence" OR "RAI" OR "Artificial Intelligence ethics" OR "ethical frameworks" OR "AI ethics" OR "trustworthy AI" OR "Explainable AI") | 4,021 |
| EBSCO | "Ethical issues" AND "ethical challenges" AND "responsible artificial intelligence" OR "trustworthy ai" OR "explainable artificial intelligence" | 1,604 |
| Web of Science | (((((((((TS=("ethical issues")) OR TS=("ethical challenges") AND TS=("Responsible AI"))) OR TS=("Ethical frameworks"))) OR TS=("ethical dilemma")))) AND TS=("AI ethics")) | 84 |
| ACM Digital Library | [Title: ethical issues] OR [[Title: ethical challenges] AND [Title: and responsible artificial intelligence] AND [Abstract: ethical frameworks] AND [Title: artificial intelligence ethics] AND [Title: explainable ai] AND [Title: trustworthy ai]] AND [E-Publication Date: (01/01/2013 TO 31/08/2023)] | 2,274 |
| ProQuest (Arts & Humanities) | abstract(Artificial Intelligence) OR abstract(Machine Learning) OR abstract(deep learning) OR abstract(neural network) AND title(Responsible Artificial Intelligence) OR title(Ethics) OR title(ethical frameworks) OR title(trustworthy) OR title(explainable) | 4,039 |
